# Exopolysaccharide from *Lactobacillus rhamnosus* KL37 Inhibits T Cell-dependent Immune Response in Mice

**DOI:** 10.1007/s00005-020-00581-7

**Published:** 2020-05-25

**Authors:** Bernadeta Nowak, Małgorzata Śróttek, Marta Ciszek-Lenda, Anna Skałkowska, Andrzej Gamian, Sabina Górska, Janusz Marcinkiewicz

**Affiliations:** 1 Department of Immunology, Jagiellonian University Medical College, Czysta 18, 31-121 Kraków, Poland; 2 Laboratory of Medical Microbiology, Ludwik Hirszfeld Institute of Immunology and Experimental Therapy, Polish Academy of Sciences, Wrocław, Poland; 3 Laboratory of Microbiome Immunology, Ludwik Hirszfeld Institute of Immunology and Experimental Therapy, Polish Academy of Sciences, Wrocław, Poland

**Keywords:** Exopolysaccharide, *Lactobacillus rhamnosus*, Immunomodulation, Collagen-induced arthritis, Inflammation, T cells

## Abstract

Exopolysaccharides (EPSs), major components of the bacterial biofilm, display strong strain-specific immunomodulatory properties. Previously, we have shown that crude EPS derived from *Lactobacillus rhamnosus* KL37 depresses the production of arthritogenic anti-collagen IgG and ameliorates collagen-induced arthritis (CIA) in DBA/1 mice, when lipopolysaccharide (LPS) was used as adjuvant. In this study, we used highly purified EPS from *L. rhamnosus* KL37 (EPS-37) to verify its anti-inflammatory properties and the ability to suppress T cell-dependent humoral response. We have employed the model of active CIA, in which mice immunized with type II collagen (CII) along with LPS were treated with pure EPS-37. Intravenous administration of purified EPS-37 markedly ameliorated arthritis and reduced CII-specific antibody production. EPS-37 injected subcutaneously reduced the clinical symptoms of CIA but without the reduction of arthritogenic antibodies. In addition, the effect of EPS-37 on T-cell functions was tested ex vivo and in vitro*.* EPS-37 inhibited the in vitro proliferation of T cells activated both in vivo (CII immunization) and in vitro (antigen/mitogen), and markedly reduced the production of interferon (IFN)-γ. These results together with other reports suggest that anti-inflammatory potential of EPS-37 depends on its ability to inhibit either one or the other or both possible inflammatory signaling pathways. Namely, Th1 → IFN-γ → M1 inflammatory macrophages → arthritis and/or Th1 → IFN-γ → B cells → arthritogenic antibodies → arthritis. We suggest that *L. rhamnosus* KL37 EPS might be utilized to control T cell-dependent immune responses in various inflammatory diseases. However, the most effective route of EPS-37 administration needs to be tailored for a given disorder.

## Introduction

Exopolysaccharides (EPSs) are major components of the bacterial biofilm matrix (Flemming et al. 2007; Maunders and Welch 2017). EPS, due to the extreme structural heterogeneity and being generated by both pathogenic and probiotic bacteria, plays various roles in our body (Castro-Bravo et al. 2018; Ciszek-Lenda 2011; Schiavi et al. 2018). The detrimental role of EPS in a course of infectious diseases is well documented (Clinton and Carter 2015; Gunn et al. 2016; Sharma et al. 2016). Many biofilm-forming pathogens are coated with EPS-containing capsule which prevents phagocytosis and facilitates subsequent microbial immune evasion (Leid et al. 2005; Thurlow et al. 2011). Less understood is the role of EPS of probiotic and commensal bacteria to maintain homeostasis of our body. It has been suggested that their EPS prevents pathogen colonization, reduces mucosa permeability and/or suppresses the host inflammatory response (Jones et al. 2014; Matsuzaki et al. 2015; Paik et al. 2018). Importantly, immunoregulatory properties of EPS are strongly strain specific (Ciszek-Lenda et al. 2011b; Górska et al. 2016). Moreover, it has been demonstrated that EPS from different probiotic bacteria might act via various receptors, such as Toll-like receptor (TLR)2, TLR4, C-type lectins or scavenger receptors (Castro-Bravo et al. 2019; Jones et al. 2014; Laino et al. 2016). For example, EPS from *Bacillus subtilis* induces the generation of anti-inflammatory M2 macrophages in a TLR4-dependent manner (Paynich et al. 2017); while, EPS from *Bacteroides fragilis* signals through TLR2 (Chang et al. 2017). Lactobacilli, the commonly used probiotics, might also regulate inflammatory response through interaction with TLR2 and/or TLR4 receptors (Castillo et al. 2011; Ren et al. 2016; Villena and Kitazawa 2013). Although immunomodulatory effects of probiotics require direct bacterium contact with immune cells, some reports demonstrated that EPS alone is also able to modulate the production of inflammatory mediators (Ciszek-Lenda et al. 2011a; Inturri et al. 2017; Salazar et al. 2014). However, EPS signaling pathway(s) and its specific receptor(s) remain unknown.

In our previous studies, we have investigated the immunomodulatory properties of a crude EPS isolated from *Lactobacillus rhamnosus* KL37, the selected probiotic bacteria strain. We have shown that *L. rhamnosus* KL37 EPS alters the production of inflammatory mediators by macrophages in vitro and inhibits the production of ovalbumin (OVA)-specific antibodies in mice immunized with OVA adjuvanted with lipopolysaccharide (LPS) (Ciszek-Lenda et al. 2011a; 2012). Moreover, EPS isolated from *L. rhamnosus* KL37 markedly reduced the production of collagen-specific antibodies and ameliorated collagen-induced arthritis (CIA) in mice, especially when LPS was used as an adjuvant (Nowak et al. 2012). These observations, together with other reports, suggested that immunoregulatory properties of exopolysaccharides are associated with both innate and adaptive immune responses activated in the presence of LPS (Ciszek-Lenda et al. 2012; Nowak et al. 2012; Wu et al. 2010). Therefore, further studies with highly purified EPS were necessary to exclude the effect of its contamination with other components of bacterial biofilm. The primary aim of this study was to examine the effect of EPS-37, pure *L. rhamnosus* KL37 EPS, on the development of CIA including its influence on humoral and T-cell response.

## Materials and Methods

### EPS-37 Isolation and Purification

Exopolysaccharide was obtained from *L. rhamnosus* KL37 strain isolated from the feces of the human newborns (Lipiński et al. 2003). Bacteria were grown for 48 h at 37 °C under anaerobic conditions in supplemented MRS liquid broth (Biocorp, Poland). Bacterial cells were harvested (7000 rpm, 4 °C, 15 min) and washed twice with phosphate-buffered saline (PBS) and once with MiliQ water as previously described (Górska et al. 2010; Nowak et al. 2012). Briefly, freeze-dried bacterial mass was extracted with 10% trichloroacetic acid. The crude EPS precipitated from the supernatant with cold 96% ethanol was collected (pellet), suspended in water, dialyzed and then lyophilized. The freeze-dried preparation of crude EPS dissolved in 50-mM Tris–HCl buffer, pH 7.5, containing 10-mM MgCl_2_ was treated with DNase (Sigma-Aldrich, Germany) and RNase (Sigma-Aldrich, Germany), followed by overnight treatment with *Streptomyces griseus* protease (Sigma-Aldrich, Germany). After dialysis, EPS was further purified by ion exchange chromatography on a DEAE-Sephadex A-25 column. The neutral fractions eluted with 20-mM Tris buffer, pH 8.2, and the charged fractions released with an NaCl gradient (0–2 M) in 20-mM Tris buffer, pH 8.2, were monitored at *λ* = 220 nm in a UV–VIS spectrometer and Knauer differential refractometer. The carbohydrate content was analyzed by the phenol/sulfuric acid method (DuBois et al. 1956), and the phosphate content was determined as described previously (Chen et al. 1956). The polysaccharide-containing fractions were pooled, desalted by dialysis against water, lyophilized, and further purified by gel permeation chromatography on a Toyopearl HW-55S column (Tosoh Bioscience LLC). The fractions eluted with 0.1-M ammonium acetate buffer were monitored for protein contamination (UV/VIS spectrometer at *λ* = 280 nm,), and for the carbohydrate content (Knauer differential refractometer). Moreover, since the structure of EPS-37 is known, the elution profile was compared with previous spectra and the pure EPS-37 was checked on 1H NMR. The degree of polymerization, which might be the issue of all natural polysaccharides, was well characterized for EPS-37 earlier and it was repeatable (stable). The chromatography fractions obtained were uniform, and typical for pure preparations.

### Lipoteichoic Acid Isolation and Purification

Lipoteichoic acid (LTA) extraction was adapted from Morath et al. (2001). *L. rhamnosus* KL37 bacteria were grown for 48 h at 37 °C under anaerobic conditions in supplemented MRS liquid broth (Biocorp, Poland) and then harvested by centrifugation at 7000 rpm for 15 min at 4 °C. Cells were washed three times with 0.1-M Tris–HCl buffer, pH 8, then resuspended in 20 ml of 0.1-M acetate buffer, pH 4.7 and were mixed with an equal volume of *n*-butanol (POCH, Poland) for 30 min at 37 °C under agitation at 300 rpm. After centrifugation at 14,000 rpm for 15 min at 4 °C, the aqueous phase was collected and lyophilized. Purification of LTA was performed as described previously (Ryu et al. 2009). Briefly, lyophilized LTA fractions were suspended in 0.1-M ammonium acetate buffer containing 15% *n*-propanol (pH 4.7), then filtered and purified by hydrophobic-interaction chromatography on an octyl-Sepharose CL-4B column. The LTA fractions were eluted by a linear gradient of *n*-propanol, 15–35% *n*-propanol in 0.1-M ammonium acetate buffer (pH 4.7) and monitored for phosphorus content as described previously (Chen et al. 1956). Pure LTA from *L. rhamnosus* KL37 refers to as LTA-37.

### Mice

Inbred DBA/1 male mice, C57BL/6 mice and OT II OVA-transgenic mice were bred in the Animal Breeding Unit, Department of Immunology of Jagiellonian University College of Medicine, Krakow. Mice were housed 5–6 per cage and maintained under clean conventional conditions with free access to standard rodent diet and water. Mice were used at 8–10 weeks of age. The authors were granted permission for this study by the Local Ethical Committee. Experiments were conducted according to the ethical guidance of the Local Ethical Committee.

### Experimental Models

#### In Vivo: Immunization with CII—Induction and Evaluation of CIA

DBA/1 mice were immunized subcutaneously with type II collagen (CII) from chicken sternal cartilage (Sigma-Aldrich, Germany) (0.2 mg/mouse) emulsified in Complete Freund’s Adjuvant (CFA; Sigma-Aldrich, Germany) (day 0).

To measure the effect of EPS-37 on antigen-induced T-cell activity, mice were injected systemically (intravenously) with EPS-37 on days 0, 3, 5 and 7. Control mice were injected with saline. Eight days after immunization mice were euthanized and lymphoid organs (spleen, lymph nodes) were collected. Cells isolated from spleen and lymph nodes were cultured for in vitro proliferation assay as described below.

**Fig. 1:**
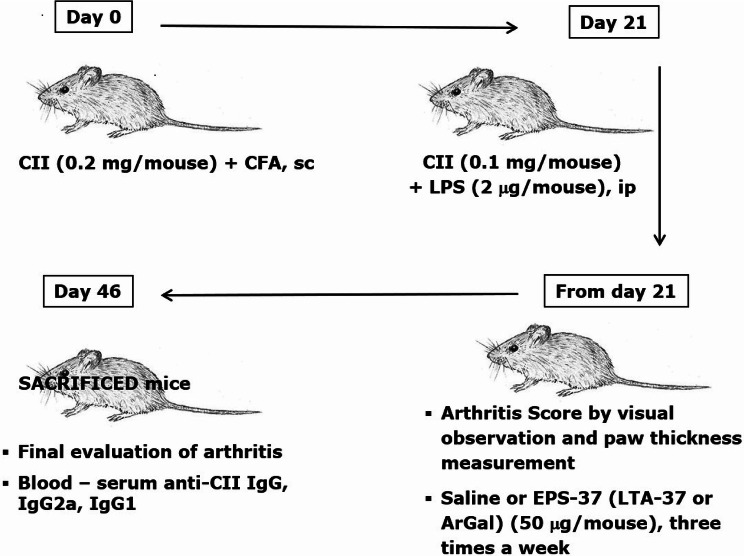
CIA induction and examination protocol: mice were immunized with CII in the presence of CFA (day 0, first immunization) and with CII in the presence of LPS (day 21, second immunization, ip: intraperitoneal). EPS-37 (or LTA-37, or ArGal) were given to mice systemically—intravenously (iv) or subcutaneously (sc) three times a week starting on the day of second immunization (day 21) till the end of the experiment. Development of arthritis was examined by visual observation, paw thickness measurement and, finally, the level of anti-CII antibodies was measured in the mouse serum

To induce CIA, 3 weeks after first immunization (on day 21), mice received boost intraperitoneal injection of CII (0.1 mg/mouse) in the presence of LPS (2 µg/mouse). EPS-37 (50 µg/mouse) was administered systemically—intravenously or subcutaneously. Injections of EPS-37 were given every other day starting on the day of the second immunization (day 21) till the end of the experiment (Fig. 1). Other cell wall component isolated from *L. rhamnosus* KL37—lipoteichoic acid (LTA-37, 50 µg/mouse), was also tested. Moss polysaccharide—arabinogalactan (ArGal, 50 µg/mouse), was used as control polysaccharide. To evaluate CIA development, mice were examined visually every other day for the incidence and severity of arthritis (joint swelling and redness). According to the increasing extend of erythema and edema of the periarticular tissues, the lesions of the four paws were each graded 0–4 as follows: 0 = no swelling/normal, 1 = slight swelling of the limb or the single digits, 2 = moderate swelling/erythema of the limb and/or multiple digits, 3 = pronounced swelling and erythema, of the limb and multiple digits, 4 = severe swelling and erythema of the limb/digits and joint rigidity/deformity. The scores of all four paws were summed to yield arthritis score (maximum 16 for each animal). Paw thickness (swelling of paws) was measured using Mitutoyo micrometer. To evaluate the level of anti-CII antibodies, the blood was collected from CIA-induced mice at the end of the experiments (day 46). Serum was prepared and stored at − 20 °C until used.

### Measurement of Serum Antigen-Specific Antibody Titers

Anti-CII antibodies in serum were measured by sandwich ELISA (Kwaśny-Krochin et al. 2002). In brief, plates (Costar EIA/RIA plates, Corning Incorporated, USA) were coated overnight with native chicken CII (5 μg/ml). 0.05% Tween 20 (Sigma-Aldrich, Germany) in phosphate buffer was used as a washing solution. Serial dilutions of mouse serum in PBS were added to antigen coated wells and incubated for 1 h at room temperature followed by biotin-conjugated antibodies against mouse immunoglobulins: IgG (Sigma-Aldrich, Germany), IgG1 (MP Biomedicals, USA) or IgG2a (Southern Biotech, USA). Horseradish peroxidase conjugated streptavidin (Vector, USA) was used as detection reagent; *o*-phenylenediamine dihydrochloride and hydrogen peroxide (both Sigma-Aldrich, Germany) were used for color development. The reaction was stopped with 3-M H_2_SO_4_ (POCH, Poland). The optical density of each sample was measured at 492 nm. The antibody levels were expressed in arbitrary ELISA units calculated from anti-CII immunoglobulin titer: 1 Unit = 1/100 titer of immunoglobulin specific to CII.

#### In Vitro: T-Cell Proliferation Assay

Cells isolated from the mouse lymph nodes or spleens were cultured in vitro in 5% CO_2_ atmosphere at 37 °C, in DMEM (Dulbecco’s modified Eagle’s medium; Lonza, Switzerland) containing 5% fecal calf serum (Biowest, France) supplemented with 25-mM HEPES (Gibco, ThermoFisher, UK), 2-mM l-glutamine (Biowest, France), 0.05-mM 2-mercaptoethanol (Gibco, ThermoFisher, UK), 5-mg/ml gentamycin (KRKA, Slovenia). Cells were seeded at 5 × 10^5^/well in 0.2 ml in 96-well plates (Falcon, Corning Inc., USA) and cultured 48 h or 72 h at 37 °C in 5% CO_2_ atmosphere. All groups were investigated in triplicates (unless otherwise stated). Cell culture supernatant was harvested (after 48 or 72 h) and stored at − 20 °C for cytokine (interferon (IFN)-γ) assay. Fresh medium was added and cells were pulsed with 3H-thymidine (1 µCi/well, Polatom, National Centre for Nuclear Research, Poland) and cultured further for 16–18 h. Then cells were harvested onto a glass fiber filter (Perkin Elmer, USA) using Filtermate Harvester (Packard Bioscience/Perkin Elmer, USA). Incorporation of radioactive 3H-thymidine was evaluated using microplate scintillation counter (Microbeta Wallac TriLux Scintillation and Luminesce Counter; PerkinElmer, USA) (Biedroń et al. 2015).

To measure (assay) the in vivo effect of EPS on CII-specific T cells, cells isolated from CII-immune, saline or EPS-37 injected, DBA/1 mice were cultured in vitro in the presence of CII (100 µg/ml).

To measure the in vitro effect of EPS-37 on T cells activated in vitro, cells obtained from C57BL/6 or OT II naïve mice were cultured in the presence of 100-μg/ml OVA or 1-µg/ml OVA 323–339 peptide or 1-µg/ml concanavalin A (ConA)—all from Sigma-Aldrich, Germany. EPS-37, pure exopolysaccharide from *L. rhamnosus* KL37 (30 µg/ml) or ArGal (30 µg/ml) (Sigma-Aldrich, Germany) were added to the culture to measure their effect on antigen (mitogen)-induced cell activation.

### IFN-γ Determination

Cell culture supernatant were harvested and stored at − 20 °C till assayed. Mouse IFN-γ ELISA Ready-SET-Go! Kit (Invitrogen, Thermo Fisher Scientific, USA) was used and the assay was performed according to the manufacturer instructions. Shortly, Costar EIA/RIA plates (Corning Incorporated, USA) were coated with anti-mouse IFN-γ antibodies overnight at 4 °C. After blocking the plates, diluted culture supernatants were applied to the plates and incubated overnight at 4 °C. Then, biotin-conjugated anti-IFN-γ antibodies were added for 1 h (room temperature), followed with avidin-HRP and TMB as detection reagent.

### Statistical Analysis

Statistical significance of differences between groups was analyzed using unpaired Student’s *t* test. Results are expressed as mean values ± SEM. A *P* value less than 0.05 was considered statistically significant (marked: *P < 0.05; **P < 0.01, ***P < 0.001). Analysis was performed using Microsoft Excel.

## Results

### Purified EPS-37, but not LTA-37, Ameliorates CIA Induced in DBA/1 Mice

We have previously shown that *L. rhamnosus* KL37 crude EPS, given to mice intraperitoneally during (and after) boost immunization, inhibits CIA development (arthritis incidence) and ameliorates severity of arthritis (arthritis score). Since peritoneal injection is impossible in humans, we have tested EPS administered intravenously or subcutaneously. Highly purified EPS-37 was used to eliminate the effect of other compounds (bacterial proteins, DNA) that might be present in the crude EPS.

**Fig. 2:**
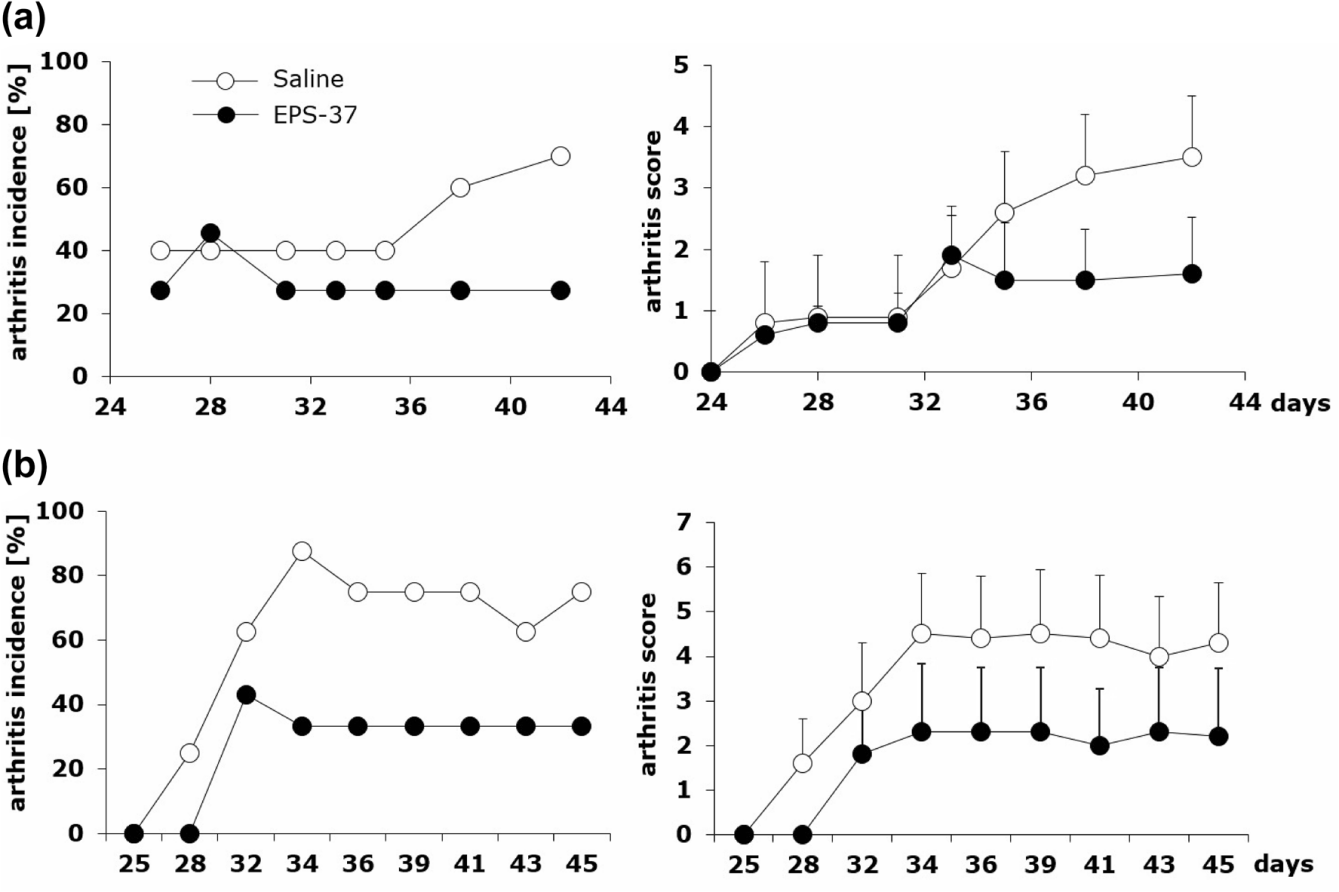
Differential effects of intravenous and subcutaneous administration of pure EPS-37 on the development of CIA. Mice immunized with CII in the presence of CFA (day 0, first immunization, sc) and with CII in the presence of LPS (day 21, second immunization, ip) were given saline (open circles) or EPS-37 (black circles) systemically, intravenously (**a**) or subcutaneously (**b**) three times a week starting on the day of second immunization (day 21) till the end of the experiment. Results are expressed as arthritis incidence—the percentage of mice with signs of arthritis and arthritis score expressed in points ± SEM. Data represent one out of three similar experiments

DBA/1 mice were immunized with CII and CFA followed with the boost immunization with CII in the presence of LPS three weeks later. On the day of second boost immunization (day 21), mice were given also EPS-37 (50 µg/mouse) systemically. EPS-37 injections were performed three times a week till the end of the experiment (day 46), as described above. As shown in Fig. 2, the number of mice developing arthritis (arthritis incidence) and the symptoms of arthritis (arthritis score) were reduced in mice given EPS-37 intravenously (Fig. 2a) or subcutaneously (Fig. 2b).

**Fig. 3:**
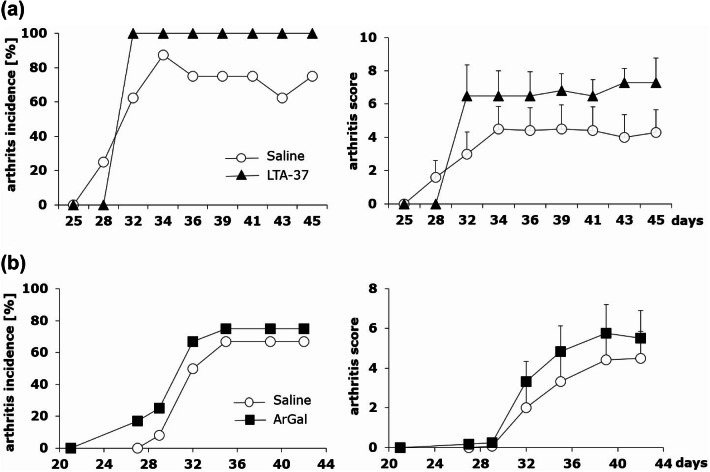
Comparison of LTA-37 and ArGal capacity to affect the development of CIA. Mice immunized with CII in the presence of CFA (day 0, first immunization, sc) and with CII in the presence of LPS (day 21, second immunization, ip) were given saline (open circles) or LTA-37 (**a** black triangles) or ArGal (**b** black squares) three times a week starting on the day of second immunization (day 21) till the end of the experiment. Results are expressed as arthritis incidence—the percentage of mice with signs of arthritis and arthritis score expressed in points ± SEM. Data represent one out of three similar experiments

Neither LTA-37, lipoteichoic acid isolated from *L. rhamnosus* KL37 (Fig. 3a), nor ArGal, moss arabinogalactan control polysaccharide (Fig. 3b), given to CII-immunized mice systemically exerted inhibitory effect on CIA development.

**Fig. 4:**
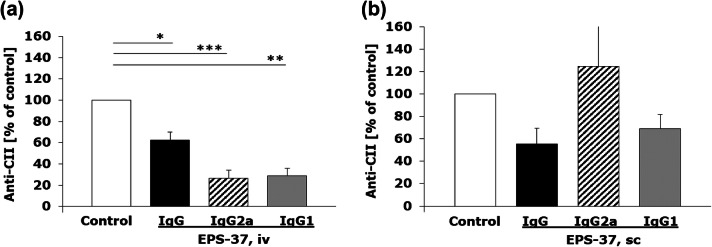
Differential effect of intravenous and subcutaneous administration of EPS-37 on the production of CII-specific IgG in the course of CIA. Mice immunized with CII in the presence of CFA (day 0, first immunization, sc) and with CII in the presence of LPS (day 21, second immunization, ip) were given EPS-37 systemically, intravenously (**a**) or subcutaneously (**b**) three times a week starting on the day of second immunization (day 21) till the end of the experiment. The level of anti-CII antibodies: IgG (black bars), IgG2a (hashed bars), IgG1 (gray bars) in serum is shown as a percentage of positive control (saline injected mice; white bars). Data represent one out of three similar experiments. Results are expressed as a mean of the measurements of each individual mouse serum ± SEM. *P < 0.05, **P < 0.01, ***P < 0.001

EPS-37 given intravenously ameliorated CIA (Fig. 2a) and significantly suppressed the production of CII-specific IgG (and IgG2a, IgG1) detected in mouse serum (Fig. 4a), as compared to control CII-immunized mice that were injected with saline. On the other hand, although EPS-37 administered subcutaneously showed suppressing effect on the development of CIA (Fig. 2b), the serum level of anti-CII IgG and IgG1 antibodies was not significantly reduced. The level of anti-CII IgG2a antibodies measured in serum was even increased, although without statistical significance (Fig. 4b).

### The Effect of EPS-37 on In Vivo Induced T-Cell Activity

**Fig. 5:**
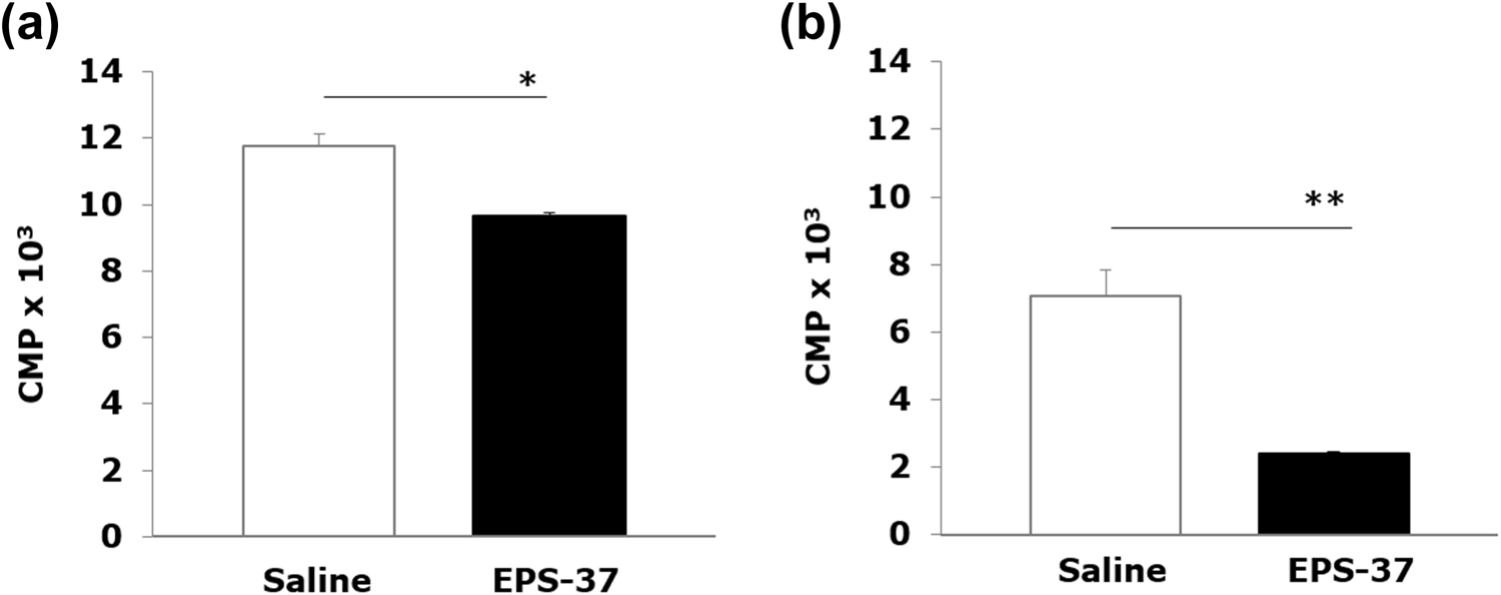
Ex vivo proliferation assay: DBA/1 mice were immunized with CII in the presence of CFA (day 0). EPS-37 (50 μg/mouse) (black bars) or saline (white bars) was injected systematically (iv) every other day (day 0, 3, 5, 7). Spleen (**a**) and lymph nodes (**b**) cells were harvested on day 8 and stimulated in vitro (5 × 10^5^/well, in 0.2 ml) with 100-μg/ml CII for 72 h. The proliferation of cells was measured by 3H-tymidine incorporation and results, counts per minute (CPM) × 10^3^, are expressed as a mean of triplicates of the culture wells ± SEM. Data represent one out of three similar experiments. *P < 0.05, **P < 0.01

Since EPS-37 affects T cell-dependent humoral response (antigen-specific antibody production, secondary immune response), we have examined the effect of EPS-37 on in vivo stimulated T cells. Antigen-specific T-cell response was tested in vitro with CII added to the culture of lymph nodes and spleen cells isolated from CII-immunized DBA/1 mice. As shown in Fig. 5, the CII-induced T-cell proliferation was suppressed when CII-immune mice were injected systemically with EPS-37.

### The Effect of EPS-37 on T-Cell Activity In Vitro

**Fig. 6:**
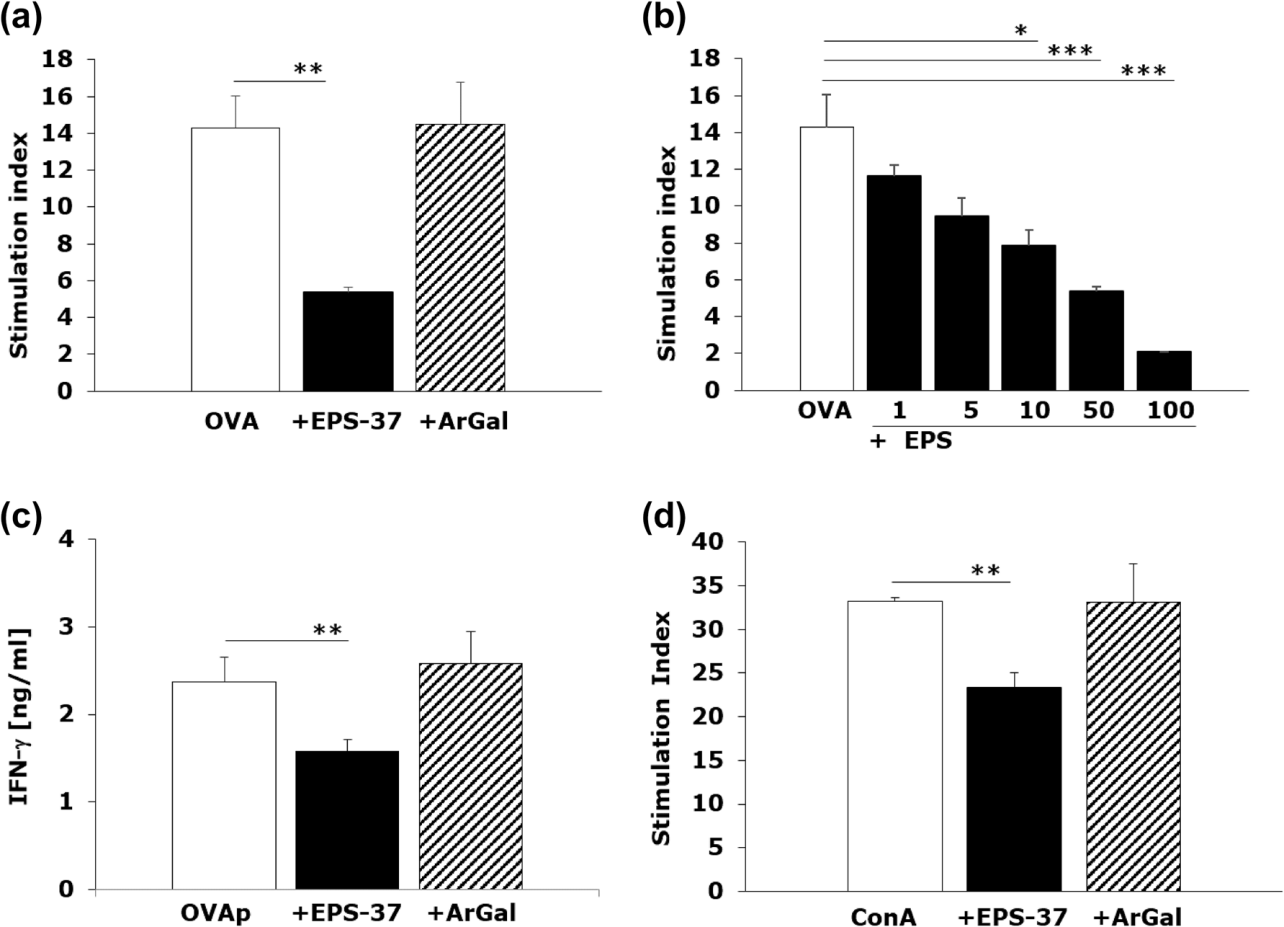
EPS-37 inhibits mitogen induced and antigen specific T-cell proliferation in vitro*.* Spleen cells from OT II transgenic (**a**–**c**) or C57BL6 mice (**d**) were stimulated (white bars) in vitro (5 × 10^5^/well, in 0.2 ml) with 100 μg/ml OVA (**a**, **b**) or 1 μg/ml OVA 323–339 peptide (OVAp, **c**), or 1 μg/ml concanavalin A (ConA, **d**). EPS-37 (black bars) (30 μg/ml; except **b** 1–100 μg/ml) or ArGal (hashed bars) (30 μg/ml) were added to the culture. 3H-thymidine was introduced into the culture for the last 18 h. The proliferation of cells is shown as stimulation index (CPM of stimulated cell/CPM of unstimulated cells) (**a**, **b**, **d**). IFN-γ (**c**) content measured in cell culture supernatant is shown in ng/ml. Results of selected experiments, expressed as a mean of triplicates of the culture wells ± SEM are shown. *P < 0.05, **P < 0.01, ***P < 0.001

Additionally, we have examined the effect of EPS-37 on in vitro stimulated T cells. Antigen-specific T-cell response was induced in vitro with OVA or OVA 323–339 peptide added to the culture of spleen cells isolated from OVA-transgenic OT II mice, in the presence of EPS-37 or ArGal. As show in Fig. 6a the OVA-induced proliferation was suppressed only when EPS-37 was introduced into the culture. The effect of EPS-37 on the proliferation of activated T cells was dose dependent as shown in Fig. 6b. Moreover, EPS-37, but not ArGal, inhibited antigen (OVA 323–339 peptide) -induced IFN-γ production (Fig. 6c). The suppressive effect of EPS-37 on T-cell proliferation was observed regardless of cell stimulation pathway. The proliferative response of cells stimulated with ConA, mitogen that triggers T cells (Palacios et al. 1982), was also inhibited when EPS-37 was added to the cell culture (Fig. 6d). Given that EPS-37, in concentrations used, was not toxic to the cells (data not shown), it was not likely that the observed effect results from cell death (apoptosis) in the presence of EPS-37.

## Discussion

In recent years, there is increasing evidence that some specific probiotic strains, including lactobacilli, are able to modulate the immune response (Lee et al. 2008; Pagnini et al. 2018; Sengül et al. 2011). Nevertheless, the mechanisms by which commensal bacteria and their products (e.g., EPS) restrict inflammation are not well understood. In this study, we have pursued our research related to immunomodulatory properties of EPS derived from *L. rhamnosus* KL37 of known chemical structure (Lipiński et al. 2003).

Previously, we have demonstrated the suppressive and anti-inflammatory potential of *L. rhamnosus* KL37-derived EPS (Ciszek-Lenda et al. 2012; Nowak et al. 2012). EPS given to CII-immunized mice systemically (intraperitoneally) resulted in an amelioration of CIA. Alleviation of clinical symptoms of arthritis was associated with the reduced production of CII-specific antibodies (Nowak et al. 2012). However, EPS used in our previous studies refers to as a crude EPS and, as a component of bacterial biofilm, may contain active amounts of other immunostimulatory macromolecules, e.g., DNA, RNA (Flemming et al. 2007). To avoid the effect of such contaminations, in the present study, we have used purified EPS-37 and its immunomodulatory properties were compared with lipoteichoic acid (LTA-37) and a control polysaccharide (ArGal).

CIA induced by immunization of DBA/1 mice with CII collagen mixed with adjuvants (CII + CFA followed by CII + LPS) was the experimental model used in this study to verify anti-inflammatory properties of EPS-37. Pure EPS-37, regardless of the routes of administration (intravenous versus subcutaneous), significantly reduced the severity and the incidence of arthritis. Neither LTA-37 isolated from *L. rhamnosus* KL37 nor ArGal affected the development of CIA indicating that anti-inflammatory properties were EPS specific. Interestingly, the amelioration of CIA was associated with the significant reduction of CII-specific antibodies, the major arthritogenic antibodies, only when EPS-37 was injected intravenously. Therefore, the mechanism(s) of beneficial effect of EPS-37 on the development of CIA without suppression of CII-specific antibody production should be explained.

It is well documented that the immune complexes (CII and anti-CII IgG) and M1 inflammatory macrophages play a central pathogenic role in CIA (Dongsheng et al. 2017). In addition, an activation of Th1 cells is necessary for the generation of CII-specific IgG (T cell-dependent humoral response) and to maintain chronic inflammation through the production of IFN-γ and tumor necrosis factor (TNF)-α (Brand et al 2003; Wang et al. 2016). Moreover, the exacerbation of CIA by LPS is associated with enhanced production of both autoantibodies (anti-CII) and inflammatory mediators, such as IFN-γ, TNF-α and IL-1 (Tanaka et al. 2013). It points to a polarization of synovial macrophages towards inflammatory M1-type cells (Li et al. 2014; Sun et al. 2017). These observations suggest various possible strategies to ameliorate arthritis in CIA: (1) inhibition of the production or neutralization of CII-specific IgG, (2) suppression of the arthritogenic pathway (Th1 → IFNγ → M1 macrophages → TNF-α) (McCann et al. 2014; Xu et al. 2017; Ye et al. 2014).

It has been experimentally proved that EPS can elicit an anti-inflammatory response when administered through several different routes (intravenous, subcutaneous, intraperitoneal) (Ciszek-Lenda et al. 2012). In our studies, both crude and purified EPS derived from *L. rhamnosus* KL37 inhibited the serum concentration of CII-specific IgG in mice. This immunomodulatory effect was associated with the decrease of both severity and incidence of CIA. Intravenous or intraperitoneal route of EPS-37 administration favors its suppressive properties due to a possible direct stimulation of spleen T regulatory cells by EPS-37 (Bermudez-Brito et al. 2018). On the other hand, other authors demonstrated that EPS from *B. subtilis* induces anti-inflammatory M2 macrophages that prevent T cell-mediated disease (Paynich et al. 2017; Paik et al. 2018). Our present results demonstrate the inhibition of T-cell proliferation and production of IFN-γ by EPS-37. It might suggest that EPS-37 is able to ameliorate the development of CIA without the reduction of anti-CII antibodies. However, the precise mechanism needs to be explained. Herein, we propose the following scenario: EPS-37 neutralizes/reduces the LPS-induced arthritogenic pathway (T cells → IFN-γ → M1 macrophages → TNF-α → inflammation → tissue destruction) by the inhibition of antigen-specific T cells.

Finally, it is important to make a point of EPS–TLR4 interactions, despite it was out of the scope of our studies. Other authors suggest that EPS is recognized by TLR4 (Castro-Bravo et al. 2019; Jones et al. 2014). Paynich et al. (2017) have shown that EPS from Gram-positive *B. subtilis* was not effective in TLR4^−/−^ mice and did not show anti-inflammatory properties. Moreover, they claim that it is unlikely that EPS functions through TLR2, similar to other structures derived from Gram-positive bacteria, because it induced M2 macrophages in TLR2^−/−^ mice. In addition, they hypothesize that EPS can bind C-type lectins and scavenger receptors, which serve as carbohydrate-binding pattern recognition receptors and can associate with TLR4 (Paynich et al. 2017).

**Fig. 7:**
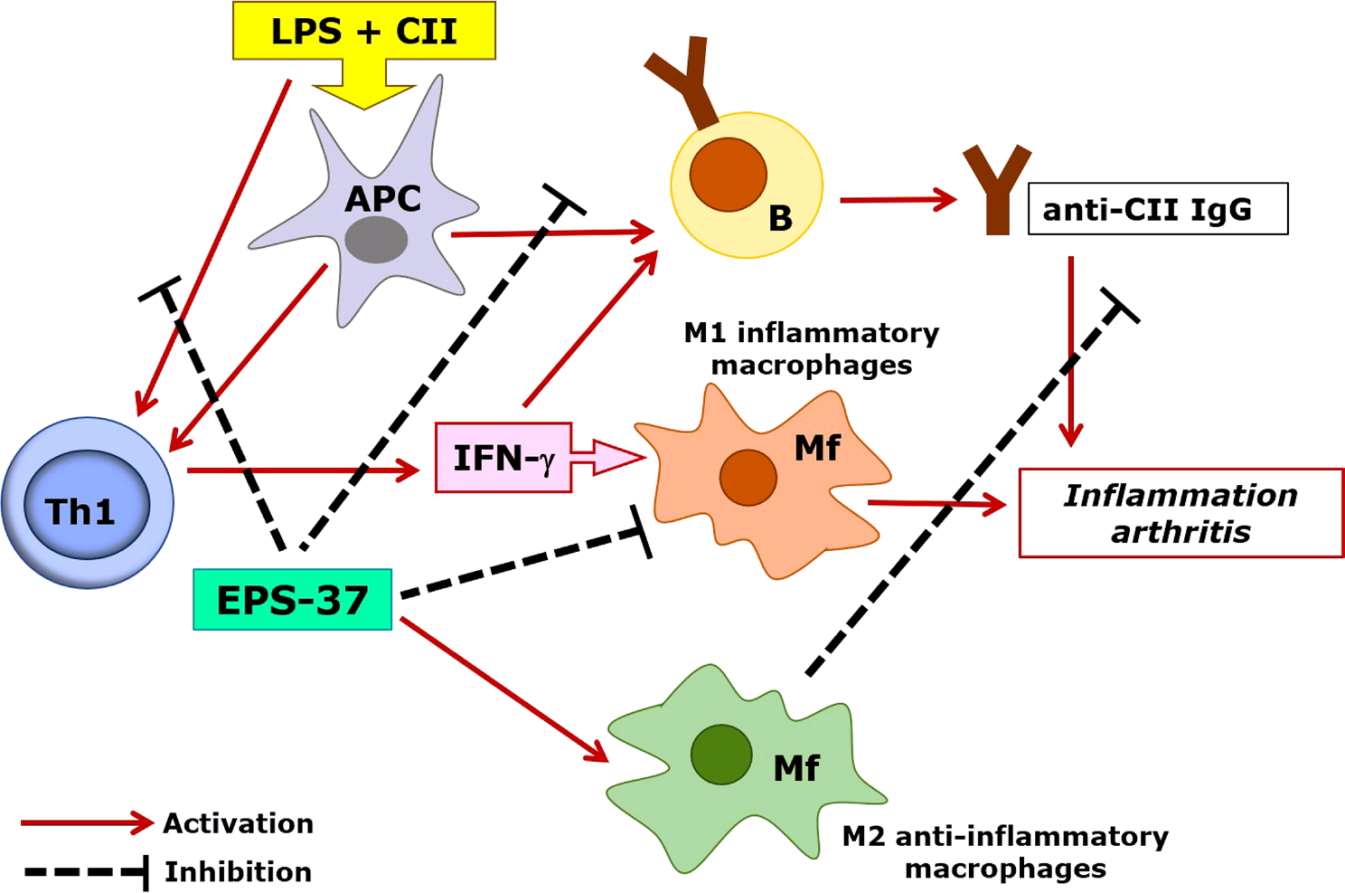
Hypothetical model of EPS-37 modulation of immune response in the course of CIA. Purified EPS-37 inhibits T-cell proliferation and a production of IFN-γ. Such action favors M2 macrophage polarization (an anti-inflammatory effect) and facilitates suppression of arthritogenic CII-specific IgG (T cell-dependent humoral response). Suppression = hash mark, Activation = solid line

In our experimental model of CIA, EPS from *L. rhamnosus* KL37 markedly ameliorated arthritis as long as it was administered simultaneously and after immunization with CII + LPS. It may suggest that EPS-37 competes with LPS for the same receptors and either reduces LPS-dependent polarization of M1 macrophages or neutralizes the LPS adjuvant effect. This yields in the induction of anti-inflammatory M2 macrophages or in the inhibition of anti-CII antibody production, respectively. On the other hand, the results from in vitro studies suggest that T cells may be the major target for EPS-37. The suppression of T cells will be responsible for both, the inhibition of T cell-dependent humoral response and the inhibition of IFN-γ production, the pivotal stimulant of M1 inflammatory macrophages (Fig. 7).

Further studies are necessary to explain precisely the mechanisms of immunomodulatory properties of EPS isolated from various bacterial species. It seems to be important as foregoing reports indicate a great anti-inflammatory therapeutic potential of EPS. However, an immunomodulatory potential of bacteria and their components (EPS, LPS) is strain specific and distinct strains of the same species may cause even opposite therapeutic effect (Górska et al. 2016; Marcinkiewicz et al. 2007). Therefore, not fully characterized EPS and applied using an incorrect route of administration may not draw the expected reaction.
